# Short-Term Cognitive and Behavioral Changes After Low-Intensity Transcranial Ultrasound Stimulation in Early Alzheimer’s Disease: An IPTW-Adjusted Retrospective Exploratory Comparative Study

**DOI:** 10.3390/brainsci16090913

**Published:** 2026-08-27

**Authors:** YoungSoon Yang, Jaeho Kim, Youngsuep Kang, Seungho Choi, Jeonghyeon Seo, SangYun Kim

**Affiliations:** 1 Department of Neurology, Soonchunhyang University College of Medicine, Cheonan Hospital, Cheonan 31151, Chungcheongnam-do, Republic of Korea; 2 Department of Neurology, Hallym University College of Medicine, Dongtan Sacred Heart Hospital, Hwaseong 18450, Gyeonggi-do, Republic of Korea; 3 Seoul National University Bundang Hospital Healthcare Innovation Park, Seongnam 13605, Gyeonggi-do, Republic of Korea; 4 Department of Neurology, Seoul National University Bundang Hospital, Seongnam 13620, Gyeonggi-do, Republic of Korea

**Keywords:** Alzheimer’s disease, low-intensity ultrasound, glymphatic clearance, Trail Making Test, inverse probability of treatment weighting, retrospective study

## Abstract

**Highlights:**

**What are the main findings?**

- Four weeks of add-on low-intensity ultrasound stimulation was associated with greater improvement in Trail Making Test Part A performance than donepezil-based standard treatment alone in amyloid PET-positive patients with early Alzheimer’s disease.
- Trail Making Test Part B showed a favorable but less robust pattern, whereas no between-group difference was observed in short-term MMSE-II change.

**What are the implications of the main findings?**

- Processing speed, visual search, and attention-related measures may be more sensitive than global cognitive screening scales for detecting short-term functional changes after low-intensity ultrasound stimulation.
- These exploratory findings support prospective randomized, sham-controlled trials with standardized cognitive assessments and biomarker endpoints.

**Abstract:**

**Background:** Low-intensity ultrasound (LIUS) has been proposed as a non-invasive approach to modulate cerebrospinal fluid movement, glymphatic–lymphatic clearance, and amyloid-beta (Aβ) handling in Alzheimer’s disease (AD). However, clinical comparisons in amyloid biomarker-confirmed patients receiving standard pharmacotherapy remain limited, and the short-term cognitive domains most likely to change after LIUS remain uncertain. **Objective:** The objective of this study was to compare 4-week changes in cognitive function, executive function, quality of life, and neuropsychiatric symptoms between amyloid PET-positive patients receiving add-on LIUS with donepezil-based standard treatment and those receiving donepezil-based standard treatment alone. **Methods:** This retrospective exploratory comparative cohort study included amyloid PET-positive patients with mild cognitive impairment or early AD who received donepezil-based standard treatment. The treatment cohort received 4 weeks of add-on LIUS at Dongtan or Bundang centers, whereas the comparator cohort received donepezil-based standard treatment alone at Soonchunhyang. Outcomes were changes from baseline to 4 weeks in Trail Making Test A (TMT-A), Trail Making Test B (TMT-B), Mini-Mental State Examination, Second Edition (MMSE-II), Quality of Life in Alzheimer’s Disease (QoL-AD), and Neuropsychiatric Inventory (NPI). Group differences were examined using unadjusted change-score comparisons and stabilized inverse probability of treatment weighting (IPTW). **Results:** The full analysis set included 51 amyloid PET-positive participants receiving donepezil-based standard treatment: 20 who additionally received LIUS and 31 who received standard treatment alone. Compared with controls, the add-on LIUS group showed greater improvement in TMT-A completion time in unadjusted analysis (−20.0 ± 31.3 s vs. −0.1 ± 5.6 s; *p* = 0.011), and this association remained significant after IPTW adjustment. TMT-B showed a favorable but less robust pattern, with borderline findings in the primary Fail-excluded analyses and a significant result in the Fail = 300 sensitivity analysis. MMSE-II change did not differ between groups. NPI improved more in the add-on LIUS group in unadjusted analysis, although this finding was exploratory. **Conclusions:** In amyloid PET-positive patients receiving donepezil-based standard treatment, 4 weeks of add-on LIUS was associated with short-term improvement in TMT-A performance compared with standard treatment alone, while MMSE-II remained unchanged. The findings suggest a preliminary signal for processing-speed or visual-search improvement and support prospective randomized sham-controlled studies with harmonized cognitive testing and biomarker endpoints.

## 1. Introduction

Alzheimer’s disease (AD) is a progressive neurodegenerative disorder characterized clinically by a decline in episodic memory, executive function, and daily functioning, and biologically by amyloid-β (Aβ) accumulation, tau pathology, synaptic dysfunction, neuroinflammation, and neuronal loss. In recent years, the therapeutic landscape of early AD has changed substantially with the development of anti-Aβ monoclonal antibodies. Lecanemab reduced amyloid markers and produced moderately less clinical decline than placebo in early AD, while donanemab also slowed clinical progression in patients with early symptomatic AD and amyloid/tau pathology [1,2]. However, these treatments do not fully stop disease progression and are associated with practical and safety concerns, including infusion burden, monitoring requirements, amyloid-related imaging abnormalities, edema, and microhemorrhage. Therefore, additional therapeutic approaches are needed that are non-invasive, repeatable, potentially compatible with standard pharmacotherapy, and capable of targeting mechanisms beyond symptomatic cholinergic modulation.

One therapeutic concept that has gained attention is enhancement in brain waste clearance. The glymphatic and meningeal lymphatic systems are thought to participate in cerebrospinal fluid (CSF) movement, interstitial fluid exchange, and clearance of soluble metabolites, including Aβ. Experimental work has suggested that impairment of meningeal lymphatic function may aggravate Aβ accumulation and age-associated cognitive dysfunction, whereas augmentation of lymphatic drainage may represent a therapeutic target in neurodegenerative disease [3,4,5]. This concept is especially relevant to AD because impaired clearance, rather than increased production alone, may contribute to Aβ accumulation in sporadic late-onset disease. However, methods to safely manipulate CSF-interstitial fluid movement or glymphatic–lymphatic clearance in humans remain under active investigation.

Focused ultrasound (FUS) and low-intensity transcranial ultrasound stimulation have emerged as non-invasive approaches with several potentially relevant biological actions. Much of the early AD-focused literature examined FUS combined with microbubbles to transiently open the blood–brain barrier (BBB), thereby enhancing drug delivery or facilitating amyloid removal. Preclinical work showed that scanning ultrasound could reduce Aβ burden and restore memory in AD mouse models [6]. Subsequent clinical studies demonstrated that FUS-mediated BBB opening can be performed repeatedly and reversibly in patients with mild AD, with closure typically occurring within 24–48 h and without serious procedure-related adverse events in small cohorts [7,8]. More recently, a proof-of-concept study combining ultrasound-mediated BBB opening with aducanumab reported greater amyloid reduction in sonicated regions than in contralateral untreated regions, although the study included only three participants and was not designed to establish clinical efficacy [9].

In parallel with BBB-opening approaches, increasing attention has been paid to low-intensity ultrasound protocols intended to influence neural function, CSF movement, or solute transport without frank BBB disruption. This distinction is important because repeated BBB opening may not be necessary for all therapeutic applications and could raise safety concerns in older patients with AD-related vascular fragility or cerebral amyloid angiopathy. Pulsed transcranial FUS has been shown in rats to enhance CSF tracer movement without BBB disruption, behavioral abnormality, or histological injury [10]. Real-time in vivo two-photon and widefield imaging later demonstrated that transcranial FUS stimulation can enhance CSF influx and perivascular-space tracer movement, supporting a direct fluid-transport mechanism [11]. Very low-intensity ultrasound has also been reported to facilitate glymphatic influx and Aβ clearance through pathways involving TRPV4 and aquaporin-4 (AQP4), suggesting that ultrasound may influence water-channel-mediated clearance physiology [12]. These studies provide a biological rationale for testing whether repeated low-intensity ultrasound stimulation can produce measurable short-term clinical changes in patients with early AD.

Several mechanistic studies further suggest that ultrasound may influence AD pathology through multiple overlapping mechanisms. In 5XFAD mice, FUS with microbubbles enhanced soluble Aβ clearance from the brain parenchyma to CSF and deep cervical lymph nodes, indicating a brain-to-CSF-to-lymphatic disposal pathway for soluble Aβ rather than direct plaque removal alone [13]. FUS-mediated methylene blue delivery reduced neural damage and Aβ plaque burden in an APP/PS1 mouse model and increased AQP4 expression, again linking ultrasound effects to water dynamics and glymphatic physiology [14]. More recently, focused ultrasonication without additional anti-amyloid drugs was reported to reduce fibrillar and oligomeric Aβ aggregates in vitro and reduce plaque burden in ex vivo and in vivo AD model experiments [15]. These data do not prove clinical efficacy, but they support the plausibility that ultrasound-based interventions may act through clearance enhancement, mechanical effects on amyloid assemblies, microglial modulation, changes in perfusion or metabolism, or neuromodulatory network effects.

Human clinical evidence for low-intensity ultrasound in AD remains preliminary but is expanding. Jeong et al. conducted a pilot study in four AD patients receiving hippocampal low-intensity transcranial FUS after microbubble contrast injection, using sonication below the threshold for BBB opening. They found no evidence of active BBB opening on dynamic contrast-enhanced MRI, observed increased regional cerebral glucose metabolism in several cortical regions, and reported mild improvement in memory, executive, and global cognitive measures without adverse events [16]. A later randomized, double-blind, placebo-controlled pilot trial of transcranial ultrasound stimulation in mild AD enrolled nine participants and reported no serious clinical or radiographic adverse events, with cognitive stabilization or improvement signals on ADAS-cog and MMSE over follow-up, although the trial was very small and requires replication [17]. These studies suggest that low-intensity ultrasound may be feasible and safe in early AD, but they do not establish robust efficacy.

The most directly related clinical evidence for the present study is a prospective early-stage AD LIUS pilot study that enrolled 10 amyloid-positive participants who underwent 4 weeks of treatment. That study reported completion of treatment without significant adverse events, improvement in neuropsychological test performance, reduced amyloid deposition mainly among APOE ε4 carriers, no significant changes in BBB integrity, microbleeds, or brain volume, and possible MRI-derived changes in putative glymphatic activity that did not survive multiple-comparison correction [18]. Importantly, however, the study was small, single-arm, and lacked a contemporaneous standard-treatment comparator. Therefore, it remained uncertain whether short-term cognitive and behavioral changes after LIUS exceed changes expected from standard medical treatment, repeated testing, regression to the mean, or natural short-term variability.

The 4-week assessment interval reflected the available LIUS treatment protocol and was consistent with the most directly relevant prior early-stage AD LIUS pilot study, in which participants received LIUS three times weekly for 4 weeks and underwent post-treatment cognitive, MRI, and amyloid PET assessments [18]. This interval should not be interpreted as sufficient to establish disease modification or durable amyloid-related clinical benefit. Rather, the 4-week interval was intended to evaluate early functional signals that might plausibly arise from neuromodulatory effects, altered CSF or perivascular fluid movement, or short-term changes in task performance. For this purpose, the Trail Making Test (TMT) was particularly relevant because it assesses domains that may change independently of global cognitive screening scores. TMT-A primarily reflects visual search, psychomotor speed, attention, and processing speed, whereas TMT-B adds set-shifting, mental flexibility, and executive-control demands. Prior neuropsychological studies support the use of TMT measures as indicators of processing speed and executive function in older adults and in patients with MCI or dementia [19,20,21]. Because global cognitive scales such as MMSE-II may be relatively insensitive to domain-specific changes over a 4-week interval, TMT-A and TMT-B were considered appropriate exploratory outcomes for detecting short-term changes in processing speed and executive performance.

In this retrospective exploratory comparative cohort study, we compared 4-week changes in cognitive, executive-function, quality-of-life, and behavioral outcomes in amyloid PET-positive patients with mild cognitive impairment (MCI) or early AD receiving donepezil-based standard treatment, according to whether they additionally received LIUS. We first examined unadjusted change-score differences using the available raw data. Because treatment allocation was non-randomized and the treatment and control datasets came from different centers, we additionally performed inverse probability of treatment weighting (IPTW) as an adjusted analysis to assess whether the observed patterns were broadly preserved after accounting for measured baseline differences. We hypothesized that add-on LIUS would show stronger short-term signals on TMT-A and TMT-B than on MMSE-II, consistent with the possibility that processing-speed and executive-function measures may be more sensitive than global cognitive screening measures over a short observation window.

## 2. Materials and Methods

### 2.1. Study Design and Data Source

This exploratory retrospective comparative cohort study used existing patient-level data from three Korean centers. Patients in the add-on LIUS cohort were derived from Dongtan and Bundang centers, whereas patients in the standard-treatment-alone comparator cohort were derived from Soonchunhyang. Before clinical implementation of the shared protocol, investigators from the participating centers jointly reviewed and aligned the clinical and assessment procedures. Common eligibility criteria, assessment schedules, outcome definitions, and treatment-stability requirements were applied across centers to promote procedural consistency. This study included amyloid PET-positive patients with MCI or early AD who had baseline and 4-week follow-up clinical assessments. Because treatment allocation was non-randomized and the treatment and comparator cohorts were derived from different centers, the results were interpreted as exploratory and hypothesis-generating. This study was conducted in accordance with the Declaration of Helsinki and was approved by the Institutional Review Board of Soonchunhyang University College of Medicine, Cheonan Hospital on 3 July 2026 (IRB No. 2023-10-025).

### 2.2. Participants and Eligibility Criteria

Eligible patients were adults with amyloid PET-positive MCI or early AD. Diagnoses of MCI and early AD were based on established clinical criteria for MCI and AD dementia, with amyloid PET positivity required as biomarker evidence of AD pathology [22,23,24]. Patients were included if they had available baseline demographic and clinical information and at least one analyzable baseline and 4-week follow-up outcome measure. The intended study population was restricted to patients in the early clinical stage of disease. Inclusion criteria were age 55–90 years at baseline, diagnosis of MCI or early AD, amyloid PET positivity, baseline Clinical Dementia Rating of 0.5 or 1 when available, available baseline MMSE-II data, and available baseline and 4-week follow-up data for TMT-A or TMT-B. All participants received donepezil 5 mg/day throughout the 4-week observation period, with no dose escalation, reduction, initiation, or discontinuation. Exclusion criteria were major structural brain disease considered to be the primary cause of cognitive impairment, such as major stroke, intracranial hemorrhage, brain tumor, or normal-pressure hydrocephalus; addition or major dose change of medications likely to affect cognition during the observation period; exposure to other non-invasive brain stimulation or investigational interventions during the observation period; major psychiatric illness or acute delirium interfering with cognitive assessment; inability to generate analyzable TMT-A or TMT-B data after applying predefined missing-data rules; and excessive missingness precluding analysis.

### 2.3. Intervention and Comparator

All patients received donepezil-based standard medical treatment during the observation period. Patients in the treatment cohort additionally received LIUS using a commercially available device, Neuclare B-01 (DeepSon Bio, Seoul, Republic of Korea). The device name and manufacturer are provided for technical identification and reproducibility. LIUS was administered at both treatment centers using the same Neuclare B-01 device, manufacturer operating instructions, standardized treatment protocol, and transducer configuration, scalp placement, and orientation. Technical reporting of the LIUS protocol was guided by the relevant ITRUSST consensus recommendations [25] and the published technical characterization of the Neuclare platform by Jo et al. [18]. The system comprised four separate unfocused single-element transducers operating at a basic frequency of 250 kHz. Each transducer had a radius of curvature of 168 mm and an aperture diameter of 43 mm. The protocol used a pulse duration of 100 ms, a pulse-repetition frequency of 1 Hz, and a duty cycle of 10%. Each session comprised 30 min of sonication. The peak acoustic pressure measured in free water was 0.39 MPa at an axial distance of 50 mm from the center of the transducer. In the previously published CT-based acoustic simulation of the same device platform, the mean estimated in situ peak pressure was 0.28 MPa, and the estimated spatial-peak pulse-average intensity (I_SPPA), spatial-peak temporal-average intensity (I_SPTA), and mechanical index were 2.6 W/cm^2^, 0.26 W/cm^2^, and 0.56, respectively [18]. These values represent the previously published technical characterization of the device platform and were not interpreted as patient-specific intracranial measurements for every participant in the present cohort. Two transducers were positioned over the frontal scalp and one over each temporal region. The frontal transducers were oriented toward the posterior fontanelle, and the temporal transducers were oriented toward the contralateral side. This standardized transducer arrangement was intended to provide broad bilateral frontal and temporal exposure rather than individualized stimulation of a stereotactically defined brain target. Because the protocol did not use participant-specific stereotactic targeting, no participant-specific intracranial target depth was prescribed. Ultrasound gel pads were used for acoustic coupling without hair removal, and the transducers were secured using a dedicated headband. LIUS was administered three times weekly for 4 weeks (12 sessions). Individualized neuronavigation, subject-specific acoustic simulation, and skull-thickness-based correction were not used for treatment delivery. Patients in the comparator cohort received donepezil-based standard medical treatment alone and did not receive LIUS during the observation period. The comparator cohort was used to estimate short-term changes observed under standard medical treatment alone and repeated neuropsychological testing.

### 2.4. Outcomes

Cognitive, executive-function, quality-of-life, and behavioral assessments were extracted at baseline and 4-week follow-up using common assessment schedules and outcome definitions across centers, although detailed information regarding examiner blinding and retest procedures was not consistently available because of the retrospective design. The co-primary outcomes were changes in TMT-A and TMT-B completion times. Change was calculated as the 4-week follow-up value minus the baseline value. For TMT-A and TMT-B, negative change values indicated shorter completion time and were interpreted as improvement. TMT-A was interpreted primarily as a measure of visual search, attention, psychomotor speed, and processing speed. TMT-B was interpreted as a more demanding measure that additionally requires set-shifting, working memory, and executive control. Because TMT-B is more difficult than TMT-A and more vulnerable to non-completion, TMT-B results were interpreted together with a sensitivity analysis for Fail values. The main secondary cognitive outcome was the change in MMSE-II score, which was included as a global cognitive screening measure based on the MMSE framework and its second edition [26,27]. For MMSE-II, positive change values indicated improvement. Exploratory outcomes included changes in QoL-AD, a disease-specific quality-of-life measure for cognitively impaired older adults [28], and NPI, a structured measure of neuropsychiatric symptoms in dementia [29]. For QoL-AD, positive change values indicated improvement. For NPI, negative change values indicated improvement in neuropsychiatric symptoms.

### 2.5. Analysis Sets and Handling of Fail Values

The full analysis set included all eligible patients with at least one analyzable baseline and 4-week follow-up outcome. The LIUS dataset initially contained 23 records. Three Bundang records were excluded because major outcome data were insufficient. The final full analysis set therefore included 20 add-on LIUS patients and 31 comparator patients. For the TMT-A analysis, 20 add-on LIUS patients and 31 comparator patients had analyzable change values. For TMT-B, Fail values were treated as missing in the primary analysis because exact completion times were unavailable. Under this rule, 18 add-on LIUS patients and 31 controls had numeric change values. In a sensitivity analysis, 300 s was assigned at each visit recorded as Fail as a pragmatic fixed-value rule.

The IPTW analysis required complete data for age, sex, baseline MMSE-II, baseline TMT-A, and baseline TMT-B. In a sensitivity analysis, the single missing age value was replaced with the cohort median of 71 years, and the propensity-score and outcome models were refitted. In the primary Fail-excluded IPTW dataset, 17 add-on LIUS patients and 31 comparator patients were analyzable. In the Fail = 300 sensitivity dataset, 19 add-on LIUS patients and 31 comparator patients were analyzable.

### 2.6. Statistical Analysis

Continuous variables were summarized as mean ± standard deviation, and categorical variables as counts and percentages. Baseline between-group differences were assessed descriptively. Within-group changes from baseline to 4 weeks were tested using paired t-tests. Unweighted between-group differences in change values were tested using Welch t-tests. Because this was an exploratory study with a small sample size, *p* values were interpreted as indicators of signal strength rather than as confirmatory hypothesis tests. Propensity scores were estimated using logistic regression with treatment status as the dependent variable and age, sex, baseline MMSE-II, baseline TMT-A, and baseline TMT-B as covariates [30,31]. Stabilized IPTW was used as an adjusted analysis to account for measured baseline differences between groups [32]. These covariates were selected because they were measured before the treatment period and were clinically relevant to both treatment assignment and outcome change. Follow-up values and change scores were not included in the propensity score model. Clinical site was not included as an adjustment variable because treatment status did not vary within site: Dongtan and Bundang contributed only LIUS-treated participants, whereas Soonchunhyang contributed only controls. Consequently, treatment and site effects could not be estimated separately; site fixed effects were perfectly collinear with treatment status, and a site random-effects model was not considered reliable with only three sites and no within-site variation in treatment exposure. Stabilized weights were calculated to reduce variance instability [32]. Covariate balance before and after weighting was evaluated using standardized mean differences and displayed in a Love plot. Effective sample size was calculated to describe the amount of information retained after weighting. Weighted mean changes were estimated for each outcome. IPTW-adjusted linear regression models were then fitted using robust standard errors. Covariate-adjusted IPTW models included the same baseline covariates used in the propensity score model to reduce residual imbalance in this small dataset. The primary TMT-B analysis excluded Fail values. The sensitivity analysis assigned Fail values to 300 s and repeated the propensity score and outcome models. No formal multiplicity adjustment was applied because this study was exploratory. However, the co-primary status of TMT-A and TMT-B was considered during interpretation; therefore, a robust TMT-A finding with a less robust TMT-B finding was not interpreted as definitive evidence of broad executive-function efficacy. Confidence intervals were reported when available. Descriptive post hoc power and 80% minimal detectable differences were calculated for the co-primary Welch comparisons using the observed group standard deviations and a two-sided α of 0.05.

## 3. Results

### 3.1. Study Population and Baseline Characteristics

The full analysis set included 51 amyloid PET-positive participants receiving donepezil-based standard treatment: 20 patients who additionally received LIUS and 31 standard-treatment-alone controls. Baseline characteristics are summarized in Table 1. The add-on LIUS cohort included 10 analyzable participants from Dongtan and 10 from Bundang; three additional Bundang records lacked sufficient major outcome data and were excluded. Site-specific baseline characteristics are presented in Supplementary Table S1; no statistically detectable differences were observed across centers, although power was limited by the small site-specific samples. Baseline age was similar between groups, although one add-on LIUS participant lacked age information (69.5 ± 8.2 vs. 71.4 ± 4.8 years). Baseline MMSE-II was numerically lower in the add-on LIUS group than in controls (22.45 ± 3.24 vs. 23.74 ± 2.19), whereas baseline TMT-A was nearly identical (115.0 ± 75.5 vs. 112.6 ± 49.6 s). Baseline TMT-B was numerically slower in the add-on LIUS group (264.2 ± 77.5 vs. 247.5 ± 85.3 s), and baseline NPI was higher and more variable in the add-on LIUS group (9.75 ± 14.72 vs. 5.45 ± 5.18).

Overall, the baseline profile did not show extreme measured imbalance, particularly for TMT-A, which was the most robust outcome in subsequent analyses. However, the numerical differences in baseline MMSE-II, TMT-B, and NPI, together with the small sample size and site-linked treatment assignment, supported the use of adjusted analyses to evaluate the robustness of the unadjusted findings.

### 3.2. Four-Week Outcome Changes After LIUS and Standard Treatment

**Figure 1:**
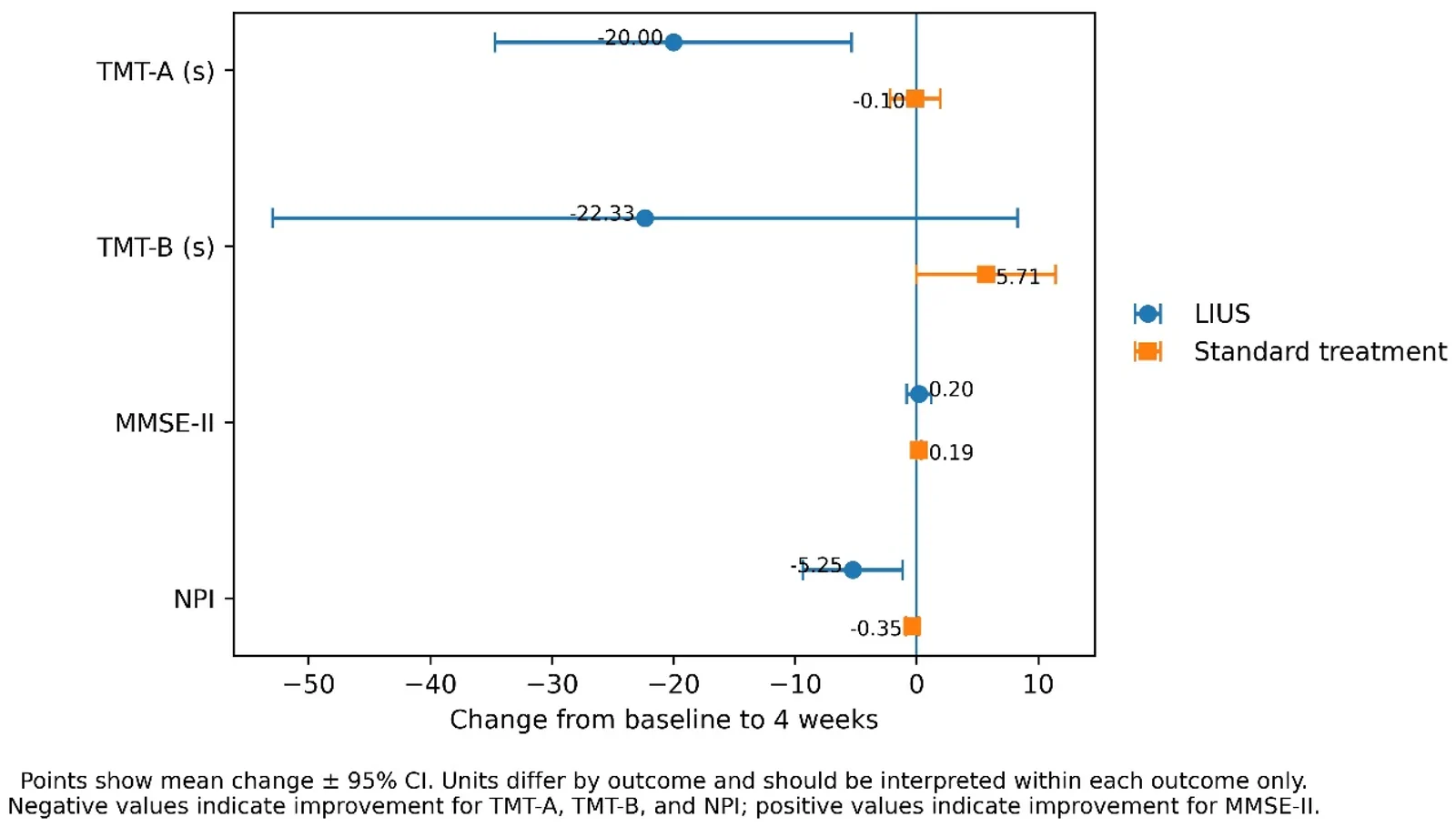
Unadjusted between-group differences in four-week outcome changes. Points represent mean differences in change between the LIUS and standard-treatment groups (LIUS minus standard treatment), and error bars indicate 95% confidence intervals. Negative values favor LIUS for TMT-A, TMT-B, and NPI, whereas positive values favor LIUS for MMSE-II. Units differ by outcome and should be interpreted within each outcome only. The primary TMT-B analysis excluded Fail values. LIUS, low-intensity ultrasound; MMSE-II, Mini-Mental State Examination, Second Edition; NPI, Neuropsychiatric Inventory; TMT-A/B, Trail Making Test Parts A and B.

Changes from baseline to 4 weeks are summarized in Table 2 and illustrated in Figure 1. TMT-A completion time improved significantly after add-on LIUS, with a mean change of −20.00 ± 31.34 s (*p* = 0.010), whereas the standard-treatment-alone control group showed almost no change (−0.10 ± 5.59 s; *p* = 0.924). The between-group difference in TMT-A change was −19.90 s (95% CI, −34.68 to −5.13; *p* = 0.011), favoring add-on LIUS. TMT-B also showed a favorable direction after add-on LIUS, although the primary Fail-excluded result was less robust. Mean TMT-B change was −22.33 ± 61.60 s in the add-on LIUS group and +5.71 ± 15.61 s in controls, yielding a between-group difference of −28.04 s (95% CI, −59.08 to +2.99; *p* = 0.074). The large variability in TMT-B change supported the need for sensitivity analysis according to Fail-value handling. Post hoc power was 76% for TMT-A and 44% for TMT-B; the corresponding 80% minimal detectable differences were 20.9 and 43.8 s. MMSE-II change was almost identical between groups (+0.20 ± 2.17 vs. +0.19 ± 0.54 points; *p* = 0.990), suggesting no measurable short-term difference in global cognitive screening performance. QoL-AD improved slightly in both groups without a significant between-group difference. NPI decreased more after add-on LIUS than in controls (−5.25 ± 8.78 vs. −0.35 ± 1.47; *p* = 0.023), although this exploratory finding should be interpreted cautiously because baseline NPI was higher and more variable in the add-on LIUS group. In an exploratory analysis, TMT-A and TMT-B changes did not differ significantly between the two LIUS centers (Supplementary Table S2).

### 3.3. Robustness of Four-Week Changes After IPTW Adjustment

The primary IPTW dataset for the Fail-excluded analysis included 48 participants: 17 add-on LIUS patients and 31 controls. Propensity scores ranged from 0.128 to 0.780, indicating partial but incomplete overlap between groups. Stabilized weights ranged from 0.454 to 2.758, with no evidence of extreme weight instability. The effective sample size was approximately 39.1, reflecting the limited information content of the weighted sample. Weighting improved measured covariate balance. The largest absolute standardized mean difference decreased from 0.585 before weighting to 0.187 after weighting, although some residual imbalance remained because of the small sample size and limited overlap. After weighting, four of the five covariates had absolute SMDs below 0.10, while baseline MMSE-II retained modest residual imbalance (SMD = 0.187; Supplementary Figure S1). IPTW-adjusted treatment effect estimates are shown in Table 3. After IPTW adjustment, the pattern of results was broadly consistent with the unadjusted four-week change analysis. The strongest signal remained TMT-A, for which add-on LIUS was associated with greater improvement in the covariate-adjusted IPTW model (β = −20.08 s; 95% CI, −39.36 to −0.79; robust *p* = 0.041). TMT-B showed a favorable but borderline association after IPTW adjustment (β = −27.21 s; 95% CI, −55.63 to +1.20; *p* = 0.061). MMSE-II and QoL-AD remained non-significant. NPI remained directionally favorable, but the robust confidence interval crossed zero (β = −4.54; 95% CI, −10.28 to +1.20; *p* = 0.121). Median-age imputation yielded similar point estimates for TMT-A (β = −18.50 s, *p* = 0.058) and TMT-B (β = −22.82 s, *p* = 0.132) without materially changing the overall pattern. Overall, the IPTW-adjusted analysis supported the same general pattern as the unadjusted analysis: TMT-A was the most robust clinical endpoint, TMT-B was favorable but less certain, and NPI represented an exploratory behavioral signal rather than a definitive outcome.

### 3.4. Sensitivity Analysis for TMT-B Fail Values

The sensitivity analysis assigning TMT-B Fail values to 300 s is also presented in Table 3. In this analysis, the IPTW sensitivity dataset included 19 add-on LIUS patients and 31 controls. Stabilized weights ranged from 0.482 to 3.395, and the effective sample size was approximately 40.2. Under this rule, add-on LIUS was associated with greater TMT-B improvement in the IPTW covariate-adjusted model (β = −32.46 s; 95% CI, −58.75 to −6.16; robust *p* = 0.016). This sensitivity result supports a favorable direction for TMT-B but also confirms that conclusions about TMT-B are sensitive to how failures are handled. Individual TMT-B change distributions under both Fail-handling rules are shown in Supplementary Figure S2. The LIUS group showed substantially greater dispersion and several extreme negative change values, supporting cautious interpretation of the mean-based estimates.

### 3.5. Safety and Tolerability

No serious adverse events or treatment discontinuations attributed to LIUS were documented during the 4-week treatment period. However, adverse events were identified retrospectively from available clinical records rather than through standardized prospective monitoring; therefore, minor or transient symptoms may have been under-recorded.

## 4. Discussion

In this retrospective exploratory comparative study of amyloid PET-positive patients receiving donepezil-based standard treatment, 4 weeks of add-on LIUS was associated with greater improvement in TMT-A completion time compared with standard treatment alone. TMT-B showed a favorable but less robust pattern, whereas MMSE-II change did not differ between groups. NPI also improved more in the add-on LIUS group in the unadjusted analysis, although this finding should be interpreted as exploratory. IPTW-adjusted analyses were broadly consistent with the unadjusted full analysis, with the most robust signal observed for TMT-A. Taken together, the results suggest a short-term association between add-on LIUS and TMT-A performance, whereas no measurable between-group difference was detected on MMSE-II over 4 weeks; the latter should not be interpreted as evidence of domain specificity.

The strongest and most reproducible finding was the improvement in TMT-A. This is clinically and methodologically important because TMT-A is a timed measure and may detect changes in processing speed and visual scanning that are not captured by global scales such as MMSE-II. Over a 4-week interval, a global cognitive instrument may be relatively insensitive because it is constrained by ceiling effects, limited item range, and relatively coarse scoring. Accordingly, the unchanged MMSE-II result should be interpreted as a null finding within this short observation window rather than as evidence that LIUS has no effect on global cognition or that its effects are specific to processing-speed measures. By contrast, timed executive or processing-speed tasks may detect smaller short-term changes in attention, psychomotor speed, arousal, visual search strategy, or task efficiency. The present finding is therefore consistent with the hypothesis that LIUS may exert early measurable effects on functional cognitive performance before global cognition changes are detectable. However, because TMT-A is also vulnerable to practice effects, attention fluctuation, examiner factors, and test familiarity, the result cannot be interpreted as definitive evidence of disease modification.

The TMT-B result requires more cautious interpretation. Although the direction favored add-on LIUS, the unadjusted comparison was borderline and the result was sensitive to handling of Fail values. This is not surprising because TMT-B is more difficult than TMT-A, particularly in patients with MCI or early AD, and completion failure or extreme values can heavily influence mean change in small samples. TMT-B also reflects a more complex combination of processing speed, working memory, set-shifting, sequencing, and executive control. Thus, TMT-B may be clinically meaningful but statistically unstable in small exploratory datasets. In future prospective studies, maximum time, discontinuation, and Fail-handling rules should be specified in advance, and executive-function measures with higher completion rates, such as the Color Trails Test or Digit Symbol Substitution Test, should be considered.

The null MMSE-II finding should not be interpreted as evidence for domain specificity. It indicates only that no measurable between-group difference was detected on this global cognitive screening measure over the 4-week observation period. This pattern is compatible with earlier low-intensity ultrasound studies that reported domain-specific or preliminary cognitive changes rather than large global cognitive effects. Jeong et al. reported mild improvements in memory, executive, and global cognitive measures after low-intensity tFUS, but the study included only four participants and lacked a control group [16]. Fuh et al. reported a small randomized placebo-controlled trial in mild AD showing safety and possible cognitive preservation, including MMSE benefit at later follow-up, but only nine participants were enrolled [17]. Jo et al. reported cognitive improvement after 4 weeks of LIUS in amyloid-positive early AD participants, but that study was single-arm and therefore could not distinguish treatment effects from repeated testing or expectancy effects [18]. Nestor et al. found no significant cognitive change but reported NPI improvement after image-guided scanning ultrasound [33], which differs from our TMT-A signal but partly accords with our unadjusted NPI improvement; however, substantial differences in study design, targeting, ultrasound protocol, and follow-up limit direct comparisons across pilot studies. The present study adds an amyloid PET-positive, donepezil-treated standard-treatment-alone comparator and suggests that the most consistent short-term signal may lie in timed executive or processing-speed measures rather than MMSE-type global screening.

The biological interpretation of these findings remains speculative. One hypothetical pathway involves altered CSF or interstitial fluid movement. Yoo et al. showed that pulsed transcranial FUS increased CSF tracer movement in rats without BBB disruption or tissue injury, supporting the possibility of acoustic-streaming-related solute transport [10]. Choi et al. provided real-time in vivo imaging evidence that transcranial FUS can increase CSF influx and perivascular-space tracer movement, suggesting a direct effect on perivascular fluid dynamics [11]. In AD model mice, FUS with microbubbles increased soluble Aβ movement from brain to CSF and deep cervical lymph nodes, and blockade of deep cervical lymphatic drainage modified the effect, supporting a glymphatic–lymphatic clearance pathway [13]. These preclinical studies suggest that non-invasive ultrasound may influence the clearance of soluble metabolites that could affect synaptic or network function.

A second possibility is that LIUS acts through neuromodulation rather than, or in addition to, clearance. Low-intensity transcranial ultrasound can modulate neural activity, although the intracranial distribution of acoustic exposure depends on transducer configuration, skull anatomy, and the targeting method. Unlike the MRI-planned, Brainsight neuronavigation-guided scanning ultrasound protocol of Nestor et al., which targeted the bilateral precuneus/posterior cingulate and temporoparietal association cortices, the present protocol used fixed, unfocused single-element transducers with standardized scalp placement intended to provide broad bilateral frontal and temporal exposure [33]. Thus, the two approaches differ substantially in focality, targeting precision, and anatomical coverage, and direct comparisons should be made cautiously. Prior human and animal studies suggest that ultrasound may influence neuronal excitability, functional connectivity, neurovascular coupling, BDNF-related pathways, and inflammatory responses. In the randomized pilot trial of transcranial ultrasound stimulation in mild AD, the authors interpreted their findings in the context of neuromodulation, neurotrophic signaling, cerebral blood flow, and neuroprotection [17]. Jeong et al. also observed increased regional cerebral glucose metabolism after hippocampal sonication without active BBB opening, which may be more consistent with metabolic or network modulation than with direct structural amyloid clearance [16]. The TMT-A signal in the present study could therefore reflect improved attentional efficiency, arousal, processing speed, or network-level function rather than direct amyloid removal.

A third possibility is interaction with amyloid pathology itself. Preclinical studies have shown that ultrasound can reduce amyloid burden through several potential mechanisms, including BBB-mediated antibody or drug delivery, activation of endogenous clearance pathways, microglial phagocytic responses, and possibly direct mechanical effects on amyloid aggregates. Leinenga and Götz demonstrated that scanning ultrasound reduced Aβ and restored memory in an AD mouse model [6]. Lee et al. later reported that focused ultrasonication could reduce fibrillar and oligomeric Aβ species in vitro and reduce plaque burden in ex vivo and in vivo mouse experiments without additional anti-amyloid drugs [15]. Nevertheless, amyloid reduction does not necessarily translate into immediate cognitive or behavioral improvement, and the 4-week duration of the present study is too short to infer disease modification. Therefore, the current clinical findings should be interpreted as functional short-term signals rather than evidence that LIUS reduced amyloid burden in these participants.

The NPI finding is intriguing but should be considered exploratory. Neuropsychiatric symptoms in AD can fluctuate over short periods and may be influenced by caregiver perception, environmental changes, sleep, attention, general medical status, and non-specific therapeutic contact. A reduction in NPI after add-on LIUS could reflect improved behavioral regulation, reduced apathy or irritability, improved sleep-wake physiology, or caregiver-observed functional improvement. It could also reflect regression to the mean because baseline NPI values were numerically higher and more variable in the add-on LIUS group. The present dataset is too small to determine whether LIUS has a direct behavioral effect. Future sham-controlled studies should blind caregivers to treatment allocation and report domain-level NPI changes to determine whether the behavioral signal is reproducible.

The present findings should also be interpreted in the broader context of AD treatment development. Anti-amyloid antibodies have validated Aβ clearance as a clinically relevant therapeutic pathway, but their modest clinical effect size and safety concerns leave room for adjunctive or alternative approaches. FUS-based technologies are attractive because they may be non-invasive, repeatable, and adaptable to different mechanisms: BBB opening for drug delivery, subthreshold neuromodulation, enhancement in CSF movement, or combined approaches. Clinical BBB-opening studies in AD have shown feasibility and safety in small cohorts, including reversible BBB opening and regional amyloid reduction, but these studies remain early phase and are not directly comparable to the present low-intensity non-BBB-opening treatment paradigm [7,8,9]. The current study therefore occupies a different position: it does not test drug delivery or regional BBB opening, but rather asks whether add-on LIUS is associated with short-term clinical changes beyond those seen with donepezil-based standard treatment alone and repeated testing.

A strength of the present study is the inclusion of a contemporaneously available amyloid PET-positive, donepezil-treated standard-treatment-alone comparison dataset. Prior LIUS clinical studies in AD were mostly single-arm or very small, making it difficult to distinguish treatment-related change from practice effects or natural short-term variability. By comparing add-on LIUS plus donepezil-based standard treatment with donepezil-based standard treatment alone, the present analysis provides a more clinically interpretable estimate of whether the observed changes exceed what might be expected from standard medical treatment and repeated testing alone. Another strength is the use of both raw change-score comparison and IPTW-adjusted analysis. The unadjusted analysis preserves the clinical transparency of the raw dataset, while IPTW provides a complementary assessment after accounting for measured baseline differences in age, sex, baseline MMSE-II, TMT-A, and TMT-B.

However, several limitations are important. First, this was not a randomized or sham-controlled study. Because the comparator group did not receive a sham procedure, non-specific effects related to treatment expectation and the sensory experience of LIUS could not be separated from a specific biological effect of stimulation, particularly for timed performance measures such as TMT-A. Second, because treatment allocation was fully linked to center, the treatment effect could not be statistically separated from a center effect, and center could not be included as an additional covariate without complete collinearity. Although common procedures and joint investigator training were used, unmeasured differences in referral patterns, local clinical practices, patient case mix, examiner behavior, testing environments, or follow-up conditions may have contributed to the observed between-group differences. IPTW could balance only the measured baseline covariates and could not remove these unmeasured site-level effects. Therefore, the treatment estimates should be interpreted as exploratory associations rather than causal effects. Third, the sample size was small, particularly for TMT-B, and the IPTW effective sample size was only 39.1; therefore, the weighted estimates remain imprecise and should be considered supportive rather than confirmatory. Fourth, repeated TMT testing over 4 weeks may produce practice effects, and the control group contained many unchanged values, raising the possibility of differences in test administration, data recording, or follow-up conditions. Fifth, although all participants were amyloid PET-positive, quantitative amyloid PET measures, follow-up amyloid PET, FDG-PET, glymphatic MRI markers, tau biomarkers, and blood biomarkers were not consistently available in both groups, preventing direct linkage between clinical change and biological mechanism. Sixth, NPI and QoL-AD are exploratory outcomes and should not be overinterpreted in a small non-randomized dataset. The IPTW analysis was used as a supportive robustness analysis rather than as a substitute for the unadjusted comparison. Seventh, LIUS was delivered using standardized anatomical transducer placement without individualized neuronavigation, subject-specific acoustic simulation, or skull-thickness-based correction. Therefore, interindividual variation in intracranial acoustic exposure and the precise anatomical distribution of sonication cannot be excluded.

The results have several implications for future trial design. First, TMT-A appears to be a promising short-term functional endpoint for LIUS studies in early AD, especially when the goal is to detect changes in processing speed, visual search, or attentional efficiency over weeks rather than months. Second, TMT-B remains important but requires careful protocol standardization because of failure rates and high variability. Third, MMSE-II may be useful for sample characterization and safety monitoring but may be insensitive as a 4-week efficacy endpoint. Fourth, future studies should include sham stimulation, blinded raters, standardized retest procedures, parallel or alternate TMT versions, predefined Fail handling, and repeated assessments to estimate practice effects. Fifth, future biomarker-embedded randomized sham-controlled trials should include longitudinal amyloid PET, tau biomarkers, plasma neurofilament light, and MRI-based glymphatic or CSF-flow measures to test these pathways directly. Finally, commercial device involvement should be transparently disclosed, and device parameters should be reported in sufficient detail to allow for reproducibility.

## 5. Conclusions

Among amyloid PET-positive patients receiving donepezil-based standard treatment, 4 weeks of add-on LIUS was associated with greater improvement in TMT-A performance than standard treatment alone, while MMSE-II did not differ between groups. TMT-B showed a favorable but less robust pattern, and NPI improvement was exploratory. These results are consistent with the hypothesis that LIUS may be associated with short-term changes in processing speed, visual search, attention, or executive-function-related performance, but they do not establish disease modification or amyloid clearance. The findings should be regarded as hypothesis-generating and support prospective randomized sham-controlled trials with harmonized cognitive testing and biomarker endpoints.

## Figures and Tables

**Table 1:** Baseline characteristics of the full analysis set.

Characteristic	LIUS	Control	*p* Value
Age, years	19; 69.47 ± 8.17	31; 71.39 ± 4.79	0.362
Male sex, *n* (%)	8/20 (40.0)	9/31 (29.0)	0.545
MMSE-II baseline	20; 22.45 ± 3.24	31; 23.74 ± 2.19	0.127
TMT-A baseline, s	20; 115.00 ± 75.45	31; 112.61 ± 49.62	0.901
TMT-B baseline, s	18; 264.17 ± 77.49	31; 247.55 ± 85.33	0.490
QoL-AD baseline	20; 32.35 ± 6.05	31; 30.42 ± 4.86	0.239
NPI baseline	20; 9.75 ± 14.72	31; 5.45 ± 5.18	0.222

Note: Values are *n* (%) or *n*; mean ± SD. Age was unavailable for one LIUS participant. TMT-B baseline excludes Fail values. *p* values are Welch t-tests for continuous variables and Fisher’s exact test for sex.

**Table 2:** Unweighted baseline-to-4-week changes.

Outcome	LIUS *n*	LIUS Change	Within *p*	Control *n*	Control Change	Within *p*	Between-Group Difference (95% CI)	Between *p*
TMT-A, s	20	−20.00 ± 31.34	0.010	31	−0.10 ± 5.59	0.924	−19.90 (−34.68 to −5.13)	0.011
TMT-B, s	18	−22.33 ± 61.60	0.142	31	+5.71 ± 15.61	0.051	−28.04 (−59.08 to 2.99)	0.074
MMSE-II	20	+0.20 ± 2.17	0.684	31	+0.19 ± 0.54	0.056	+0.01 (−1.02 to 1.04)	0.990
QoL-AD	20	+1.25 ± 4.36	0.216	31	+1.06 ± 2.45	0.022	+0.19 (−2.01 to 2.38)	0.864
NPI	20	−5.25 ± 8.78	0.015	31	−0.35 ± 1.47	0.190	−4.90 (−9.03 to −0.76)	0.023

Note: Change = 4-week follow-up minus baseline. For TMT-A, TMT-B, and NPI, negative values indicate improvement. For MMSE-II and QoL-AD, positive values indicate improvement. Between-group *p* values are from Welch t-tests.

**Table 3:** IPTW-adjusted treatment effect estimates.

Outcome	*n* LIUS/Control	Weighted Mean Change, LIUS	Weighted Mean Change, Control	IPTW Covariate-Adjusted β (95% CI)	Robust *p*
TMT-A, s	17/31	−18.68	−0.33	−20.08 (−39.36 to −0.79)	0.041
TMT-B, s (Fail excluded)	17/31	−22.21	+5.05	−27.21 (−55.63 to +1.20)	0.061
MMSE-II	17/31	+0.33	+0.33	+0.06 (−1.15 to +1.27)	0.923
QoL-AD	17/31	+2.28	+1.07	+1.14 (−1.87 to +4.15)	0.459
NPI	17/31	−4.50	−0.19	−4.54 (−10.28 to +1.20)	0.121
TMT-B, s (Fail = 300 sensitivity)	19/31	−31.09	+5.11	−32.46 (−58.75 to −6.16)	0.016

Note: Propensity scores were estimated using age, sex, baseline MMSE-II, baseline TMT-A, and baseline TMT-B. Stabilized IPTW was applied. β estimates represent LIUS minus control difference in change. For the Fail = 300 sensitivity analysis, TMT-B Fail values were assigned to 300 s and the propensity score model was refitted.

## Data Availability

The deidentified data and code used in this study are available from the corresponding author upon reasonable request for non-commercial research, subject to institutional review board approval and data use agreement.

## Supplementary Materials

The following supporting information can be downloaded at: https://www.mdpi.com/article/10.3390/brainsci16090913/s1, Figure S1: Covariate-balance (Love) plot before and after stabilized inverse probability of treatment weighting; Figure S2: Distribution of TMT-B changes according to Fail-handling rule; Table S1: Baseline characteristics according to clinical center; Table S2: Changes in co-primary outcomes according to LIUS treatment center.
