# Supplementary material for: Short-Term Cognitive and Behavioral Changes After Low-Intensity Transcranial Ultrasound Stimulation in Early Alzheimer’s Disease: An IPTW-Adjusted Retrospective Exploratory Comparative Study
Source: Brain Sci. 2026 Aug 27;16(9):913. doi: 10.3390/brainsci16090913 (PMC13604249; doi:10.3390/brainsci16090913)
Supplement: Supplementary file 1 [file brainsci-16-00913-s001.zip › brainsci-4494751-supplementary.pdf]

Table S1. Baseline characteristics according to clinical center.

Characteristic	Dongtan (n = 10)	Bundang (n = 10)	Soonchunhyang (n = 31)	p value
**Age, years**	69.10 ± 9.20	69.89 ± 7.39 (n = 9)	71.39 ± 4.79	0.682
**Male sex, n (%)**	2 (20.0)	6 (60.0)	9 (29.0)	0.130
**MMSE-II baseline**	21.90 ± 2.85	23.00 ± 3.65	23.74 ± 2.19	0.207
**TMT-A baseline, s**	115.60 ± 70.05	114.40 ± 84.32	112.61 ± 49.62	0.991
**TMT-B baseline, s**	272.38 ± 98.53 (n = 8)	257.60 ± 60.69	247.55 ± 85.33	0.793
**QoL-AD baseline**	29.80 ± 5.03	34.90 ± 6.12	30.42 ± 4.86	0.118
**NPI baseline**	8.70 ± 8.49	10.80 ± 19.57	5.45 ± 5.18	0.419

Data are presented as mean ± standard deviation unless otherwise indicated. Continuous variables were compared using Welch analysis of variance, and sex was compared using the Fisher-Freeman-Halton exact test. TMT-B Fail values were treated as missing.

Abbreviations: MMSE-II, Mini-Mental State Examination, Second Edition; TMT-A/B, Trail Making Test Parts A and B; QoL-AD, Quality of Life in Alzheimer's Disease; NPI, Neuropsychiatric Inventory.

Table S2. Changes in co-primary outcomes according to LIUS treatment center.

Outcome	Dongtan	Bundang	p value
**TMT-A change, s**	−28.3 ± 30.6 (n = 10)	−11.7 ± 31.4 (n = 10)	0.247
**TMT-B change, s**	−41.6 ± 60.1 (n = 8)	−6.9 ± 61.3 (n = 10)	0.246

Note. Data are presented as mean ± standard deviation. Negative changes indicate improvement. Between-center p values were calculated using Welch t-tests. TMT-B Fail values were treated as missing.

Abbreviations: LIUS, low-intensity ultrasound stimulation; TMT-A/B, Trail Making Test Parts A and B.

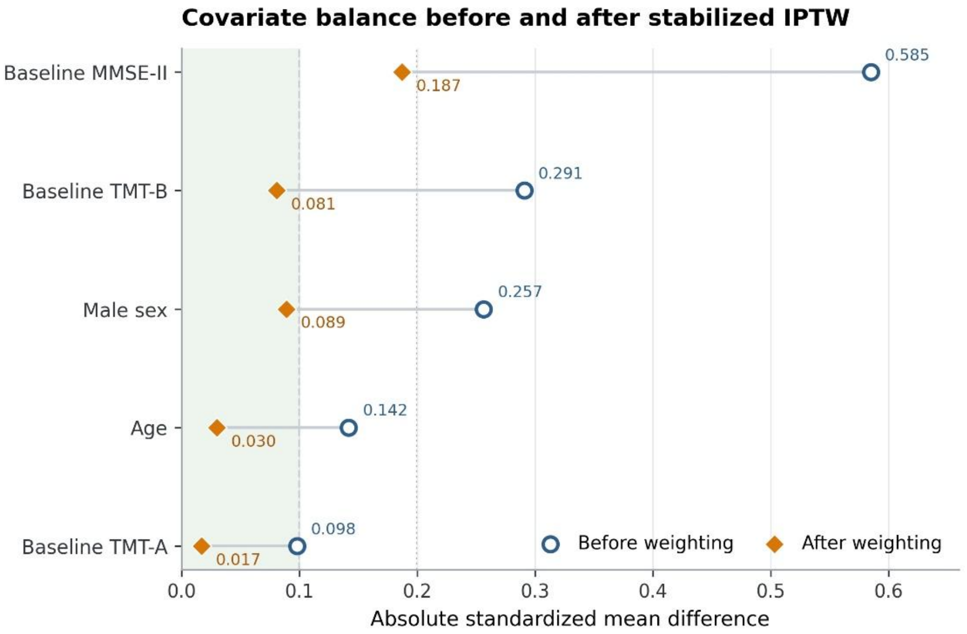

Figure S1. Covariate-balance (Love) plot before and after stabilized inverse probability of treatment weighting. Absolute standardized mean differences (SMDs) for age, sex, baseline MMSE-II, baseline TMT-A, and baseline TMT-B are shown before weighting (open blue circles) and after weighting (orange diamonds) in the primary Fail-excluded IPTW dataset (17 LIUS-treated participants and 31 controls). Values closer to zero indicate better covariate balance. The shaded area represents an absolute SMD below 0.10, and vertical reference lines are shown at 0.10 and 0.20. IPTW, inverse probability of treatment weighting; MMSE-II, Mini-Mental State Examination, Second Edition; TMT-A/B, Trail Making Test Parts A and B.

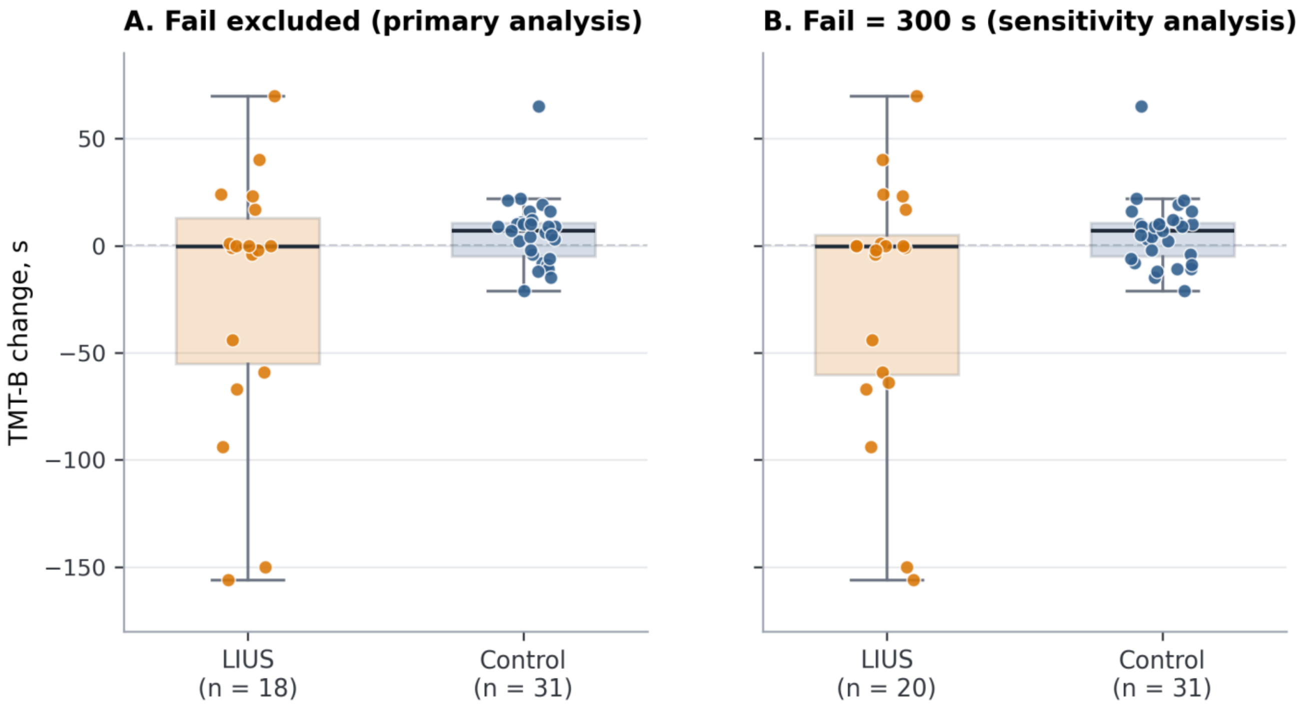

Figure S2. Distribution of TMT-B changes according to Fail-handling rule.

Individual baseline-to-4-week TMT-B changes are shown for the LIUS and control groups when Fail values were excluded from the primary analysis (A) and when 300 s was assigned at each visit recorded as Fail in the sensitivity analysis (B). Negative values indicate improvement. Points represent individual participants; boxes indicate the median and interquartile range, with whiskers extending to 1.5 times the interquartile range. The primary analysis included 18 LIUS-treated participants and 31 controls, and the Fail = 300 s sensitivity analysis included 20 LIUS-treated participants and 31 controls.

Abbreviations: LIUS, low-intensity ultrasound stimulation; TMT-B, Trail Making Test Part B.
